# Quantifying rival bond fission probabilities following photoexcitation: C–S bond fission in *t*-butylmethylsulfide†
†Electronic supplementary information (ESI) available: Room temperature near UV absorption spectrum of BSM vapour; thermochemical threshold energies for dissociative ionization of BSM and for the photodissociation of BSM^+^ cations; images of *m*/*z* 57, 41 and 29 fragments formed by pump only excitation; comparisons of the M and BS, and B and MS, momentum distributions derived from the respective images measured following photolysis of BSM at *λ* = 225.0 nm; radial distributions derived by integrating over the *m*/*z* 15 (M^+^), 47 (MS^+^) and 45 (HCS^+^) ion signals following *λ* = 225.0 nm photolysis of MSM and BSM and subsequent SPI at *λ* = 118.2 nm, along with the best-fit Gaussians that are used as the basis functions when fitting the corresponding distributions from the BSM/MSM mixtures; best-fit parameters for the Gaussian functions used to describe the M^+^, MS^+^ and HCS^+^ ion velocity distributions following photolysis of MSM and of BSM at *λ* = 227.5, 225.0 and 222.5 nm with, in each case, subsequent SPI at *λ* = 118.2 nm; harmonic vibrational wavenumbers from the ground states of M, MS, B, BS and BSM. See DOI: 10.1039/c9sc00738e


**DOI:** 10.1039/c9sc00738e

**Published:** 2019-04-23

**Authors:** Matthew Bain, Christopher S. Hansen, Tolga N. V. Karsili, Michael N. R. Ashfold

**Affiliations:** a School of Chemistry , University of Bristol , Bristol , BS8 1TS , UK . Email: mike.ashfold@bristol.ac.uk; b School of Chemistry , University of New South Wales , Sydney , NSW 2052 , Australia . Email: christopher.hansen@unsw.edu.au; c Department of Chemistry , University of Louisiana at Lafayette , Louisiana , LA 70504 , USA

## Abstract

We illustrate a new, collision-free experimental strategy that allows determination of the absolute probabilities of rival bond fission processes in a photoexcited molecule – here *t*-butylmethylsulfide (BSM). The method combines single photon (‘universal’) ionization laser probe methods, simultaneous imaging of all probed fragments (multi-mass ion imaging) and the use of an appropriate internal calibrant (here dimethylsulfide). Image analysis allows quantification of the dynamics of the rival B–SM and BS–M bond fission processes following ultraviolet (UV) excitation of BSM and shows the former to be twice as probable, despite the only modest (∼2%) differences in the respective ground state equilibrium C–S bond lengths or bond strengths. Rationalising this finding should provide a stringent test of the two close-lying, coupled excited states of ^1^A′′ symmetry accessed by UV excitation in BSM and related thioethers, of the respective transition dipole moment surfaces, and of the geometry dependent non-adiabatic couplings that enable the rival C–S bond fissions.

## Abstract 2

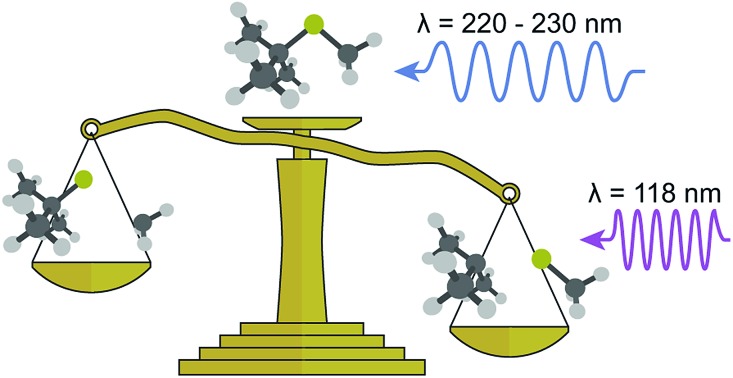
Competitive bond fission probabilities in a photoexcited molecule are quantified using contemporary ion imaging methods.

## 1 Introduction

The chemical reaction dynamics community has long aspired to predict and/or control the fission of particular bonds in a molecule. Laser excitation of selected overtones of, for example, the O–H or O–D stretch vibration in the ground electronic state of a jet-cooled sample of HOD molecules prior to ultraviolet (UV) excitation and photodissociation offers one route to achieving this goal.^1^ Such methods lack generality, however. For most polyatomic molecules, it is a challenge even to quantify the relative efficiencies of rival fragmentation channels following photoexcitation to energies above the respective dissociation limits. Experimental advances now often allow exquisitely detailed investigation of one fragmentation channel (*e.g.* a channel that yields an H atom product,^2^ or an atom or small radical amenable to resonance enhanced multiphoton ionization (REMPI) and velocity map imaging (VMI)^3^), while remaining blind to possible rival decay pathways.

An ‘ideal’ experiment for studying molecular photofragmentation processes would allow detection of all fragments of interest (*i.e.* ‘universal’ detection), while maintaining all other experimental conditions constant. This can now be achieved when, for example, photoexcitation prepares a molecular di- or trication, the decay of which yields charged particles whose velocities (and momenta) can be determined by coincidence imaging methods.^4^,^5^ Coincidence methods also enable detailed photofragmentation studies of selected radicals, where the species of interest is first prepared and accelerated as the corresponding anion. Photodetaching the electron yields the radical, which is then photodissociated using a second pulsed laser. The neutral fragments recoil from the interaction volume centred on the fast beam axis and have sufficient laboratory frame velocities to be detectable on a time and position sensitive detector.^6^ However, these challenging experiments are still far from general. Coincidence methods require low signal levels, minimal backgrounds and long acquisition times in order to acquire statistically significant results.

Extension to the photofragmentation of a closed shell neutral molecule into neutral fragments remains challenging. The development of photofragment translational spectroscopy methods, where a molecular beam containing the precursor of interest is intercepted by a photolysis laser pulse and the velocity (*i.e.* speed and angular) distributions of the products are measured by time-of-flight (TOF) methods, was an important advance. In the early experiments, universal detection of the neutral fragments relied on ionization by electron bombardment just before entering a mass spectrometer detector.^7^,^8^ Later experiments replaced the post-TOF ionization step (which was rather inefficient and prone to unwanted fragmentation) with a second laser pulse, designed to ionize the neutral fragments immediately after their formation in the interaction volume. The ionized fragments are then extracted using a carefully designed set of ion optics and their identities and recoil velocities deduced from the times and positions at which they impact on a position-sensitive detector. This is the basis of the VMI method,^9^ wherein fragment ionization is achieved by REMPI (a quantum-state specific detection method, suitable for a limited library of known products), by single photon ionization (SPI) or, recently, by strong field ionization with an intense near-infrared femtosecond laser pulse.^10^ Such VMI experiments have traditionally monitored just one product, but this limitation is increasingly being relaxed by the advent of fast-framing cameras developed specifically for chemical dynamics experiments.^11^–^13^ The latest such devices allow monitoring of more than one product channel, simultaneously (*i.e.* multi-mass detection), with high enough signal levels that experiments can be performed within the timescale of any drifting parameters.

SPI with a vacuum UV (VUV) photon^14^,^15^ is a universal detection method when the photon energy exceeds the ionisation potentials of all species of interest, and is used in the present study. While a universal ionization capability is necessary, it is not sufficient for defining the relative yields of different fragments following excitation to energies above their respective dissociation limits. For this, we also need to know the respective detection efficiencies and, ideally, would monitor all product channels simultaneously to mitigate against any short term drifts in experimental conditions (*e.g.* in the intensities of the molecular beam and/or laser pulses, or in their mutual overlap in the interaction region).

This is the achievement of the present study, wherein we determine the relative probabilities of rival S–C bond fission channels (1a) and (1b) in an asymmetric thioether, 2-methyl-2-(methylthio)-propane (henceforth *t*-butylmethylsulfide (^*t*^Bu–S–Me, or simply BSM)), following photoexcitation at UV wavelengths *λ* = 227.5, 225.0 and 222.5 nm.

1a BSM + *hν* (*λ* ∼ 225 nm) → B + SM

1b → BS + M

There are three key elements to this study. First, as Table 1 shows, all four primary photofragments (the *t*-butyl (B), methyl (M), *t*-butylthiyl (BS) and methanethiyl (MS) radicals) should be amenable to SPI with a *λ* = 118.2 nm (*hν* = 10.48 eV) photon (obtained as the 9th harmonic of the fundamental output of a Nd:YAG laser), allowing their recoil velocity distributions to be determined by VMI. Second, the required branching fractions can be determined from just the measured yields of M and MS products, the relative detection efficiencies of which are obtained by simultaneous detection of M and MS products formed, necessarily, in a 1 : 1 ratio by UV photolysis of an internal symmetric thioether calibrant: dimethylsulfide (Me–S–Me, henceforth MSM).^16^ Third, the ions formed in each photolysis/SPI cycle are detected using an event-triggered, high frame rate, Pixel Imaging Mass Spectrometry (PImMS2) sensor.^12^ This ensures that the entire 3-D velocity distributions of all ions of interest in the TOF mass spectrum are recorded in a single experiment (*i.e.* multi-mass imaging), thereby relieving the more obvious sources of experimental drift.

**Table 1:** Literature values for the ionization potentials (IPs) and enthalpies of formation (Δ_f_*H*) of BSM, MSM and selected fragment species relevant to the present work, together with B–SM, BS–M and M–SM bond strengths derived from these literature quantities. The bond strengths in parentheses are determined in the present work using the CBS-QB3 method, zero-point corrected using the harmonic normal mode wavenumbers listed in Table S4

Species	IP/eV	Δ_f_*H* (0 K)/eV
BSM	∼8.3–8.5 (ref. 43)	–1.257 ± 0.008 (ref. 44)
BS		0.44 ± 0.08 (ref. 44)
B	6.58 ± 0.01 (ref. 45)	0.782 ± 0.007 (ref. 46)
MSM	8.6903 ± 0.0009 (ref. 47)	–0.22 (ref. 48)
MS	9.262 ± 0.005 (ref. 49)	1.346 ± 0.018 (ref. 50)
M	9.843 ± 0.002 (ref. 51)	1.552 ± 0.001 (ref. 46)
S	10.360 (ref. 52)	2.873 ± 0.003 (ref. 53)

Dissociation energies (0 K)/eV	
BSM → BS + M	3.25 ± 0.08 (3.162)
BSM → B + MS	3.39 ± 0.03 (3.095)
MSM → M + MS	3.12 ± 0.02 (3.142)

Thioethers^17^–^19^ and thioanisoles^20^–^24^ are popular test-beds for exploring and illustrating the role of non-adiabatic couplings between potential energy surfaces (PESs) in the photodissociation of polyatomic molecules. The UV absorption spectrum of MSM, the prototypical thioether, shows a long wavelength onset at *λ* ∼ 240 nm, an obvious peak at *λ* ∼ 228 nm and undulatory structure to shorter wavelengths.^25^,^26^ As in the prototypical sulfide (H_2_S),^27^,^28^ theory identifies two electronic transitions in the energy range spanned by this absorption, both originating from the highest occupied molecular orbital (HOMO, the non-bonding S-centred orbital of b_1_ symmetry) and terminating on, respectively, a Rydberg-like 4sa_1_ orbital and a C–S–C antibonding orbital of b_2_ symmetry, giving excited states with respective symmetries ^1^B_1_ and ^1^A_2_ (in *C*_2v_). Excitation to the latter from the X[combining tilde] ^1^A_1_ ground state is electric dipole forbidden at *C*_2v_ geometries, but the symmetries of both excited states reduce to ^1^A′′ upon asymmetric distortion (*i.e. en route* to S–C bond fission), and excitations to both the 1^1^A′′ and 2^1^A′′ states contribute to the observed absorption. The PESs for these two states show a seam of conical intersection in the region accessed by vertical (or Franck–Condon (FC)) excitation from the X[combining tilde] state,^29^ which accounts for the prompt S–C bond fission observed following UV photolysis of MSM.^16^–^19^ The present work reveals similar fragmentation dynamics following UV excitation of BSM, quantifies the deduced preference for B–SM bond fission, and reports cuts through *ab initio* PESs for the excited states that offer a (far from complete) rationale for the observed fragmentation dynamics.

## 2. Experimental and computational methods

The apparatus and procedures have been detailed previously.^16^,^30^ Gas mixtures of BSM and MSM (both from Sigma-Aldrich, with stated purities >99%), or the respective pure samples, were prepared by drawing the vapour off liquid mixtures with respective concentrations chosen to give the correct ratio of partial vapour pressures according to Raoult's law. These ratios were then verified by Fourier transform infrared (FT-IR) spectroscopy, against quantitative calibration curves for the respective pure samples. These mixtures were then seeded in He (10% mixture, 800 mbar backing pressure), expanded through a pulsed valve into a source vacuum chamber and skimmed *en route* to the differentially pumped interaction region between the repeller and extractor plates of an ion optics assembly.^31^ Here the pulsed beam of gas (which defines the *z*-direction) was crossed by the focussed, counter-propagating (along the *y*-axis) photolysis and, *t* ∼ 20 ns later, SPI laser outputs. The former was generated by frequency doubling the output of a Nd:YAG pumped dye laser and focussing through a lens of focal length (f.l.) 25 cm positioned 20 cm in front of the molecular beam. The *λ* = 118.2 nm photons were produced by focusing (f.l. 30 cm) the third harmonic output of a second Nd:YAG laser into a phase matched Xe/Ar mixture. The resulting VUV beam (and the *λ* = 355 nm light from which it is derived) passed through a custom lithium fluoride lens (f.l. 14 cm at *λ* = 118.2 nm) such that the VUV radiation was also focussed a couple of cm beyond the interaction region. Both outputs were linearly polarized, with the electric (**ε**) vectors aligned vertically (*i.e.* along the *x*-axis). Ions formed in the interaction region were accelerated along the *z*-axis, mass separated in a field free drift region and ultimately impacted on a time and position sensitive detector (triple stack microchannel plate (MCP) detector coupled to a P47 phosphor screen) which was imaged with a PImMS2 sensor.^12^ The detector gain could be time-gated to allow selection of just a portion of the ion TOF spectrum, and the time resolution of the PImMS2 sensor was set to 25 ns which, for the employed ion optics voltages, provided 8–10 time slices through the *m*/*z* peaks of most interest.

Data processing began by centroiding the (*x*, *y*, *t*) event list from each laser shot to reduce event clusters to single ion events in time and space.^32^ TOF spectra were created at this stage, by summing all events in the various time bins and converting to the corresponding *m*/*z* spectrum. Ion images were derived by plotting the (*x*, *y*) coordinates of events within the full TOF spread associated with the *m*/*z* peak of interest (a crushed image), rather than the single time bin corresponding to the peak TOF signal (a slice image), since the latter comprises a constant slice in TOF and not in *m*/*z*. Any disproportionately intense pixel counts (from dark counts on the sensor) were discarded and set to the average value returned by the neighbouring eight pixels, after which the crushed images were reconstructed using a polar onion peeling algorithm^33^ and the recoil anisotropy parameters (*β*) of features of interest determined by fitting the angular intensity distributions to eqn (2),

2 *I*(*θ*) ∝ 1 + *β*(*P*_2_(cos *θ*))

where *P*_2_(cos *θ*) is the second order Legendre polynomial and *θ* the angle between the **ε** vector of the photolysis laser and the recoil velocity vector. Radius to velocity calibration was achieved by monitoring O^+^ signals from the well characterized photodissociation of O_2_ and REMPI of the resulting O(^3^P) atom fragments at *λ* = 225.67 nm.^34^

The ground state minimum energy geometry of the BSM molecule was optimized using Møller–Plesset second-order perturbation theory (MP2)^35^ coupled with Dunning's correlation-consistent basis set of double-zeta quality (cc-pVDZ).^36^ Relaxed potential energy cuts (PECs) along the BS–M and B–SM coordinates were computed at MP2-optimized geometries using complete active space second-order perturbation theory (CASPT2)^37^,^38^ for the single point energies with the aug-cc-pVDZ basis set.^36^ These computations were based on a state-averaged complete active space self-consistent field (SA-CASSCF) reference wavefunction. In these computations, the bond length of interest was progressively stepped and, at each step, the remaining degrees-of-freedom were allowed to relax to their minimum energy configuration. The chosen active space comprised of the three highest occupied valence orbitals (two σ orbitals and an S centred out-of-plane p-orbital) and the two lowest unoccupied (σ*) orbitals. An imaginary level shift of 0.5*E*_H_ was applied to aid convergence and to mitigate against the involvement of intruder states. Thermochemical data for the BS–M and B–SM bond energies and the parent molecular geometry were derived using the CBS-QB3 complete basis set method.^39^ The MP2, CASSCF and CASPT2 computations were undertaken in Molpro 2015,^40^ whilst the CBS computations were undertaken in Gaussian 16.^41^

## 3 Results and discussion

### 3.1 The dynamics of the C–S bond fission processes

**Fig. 1:**
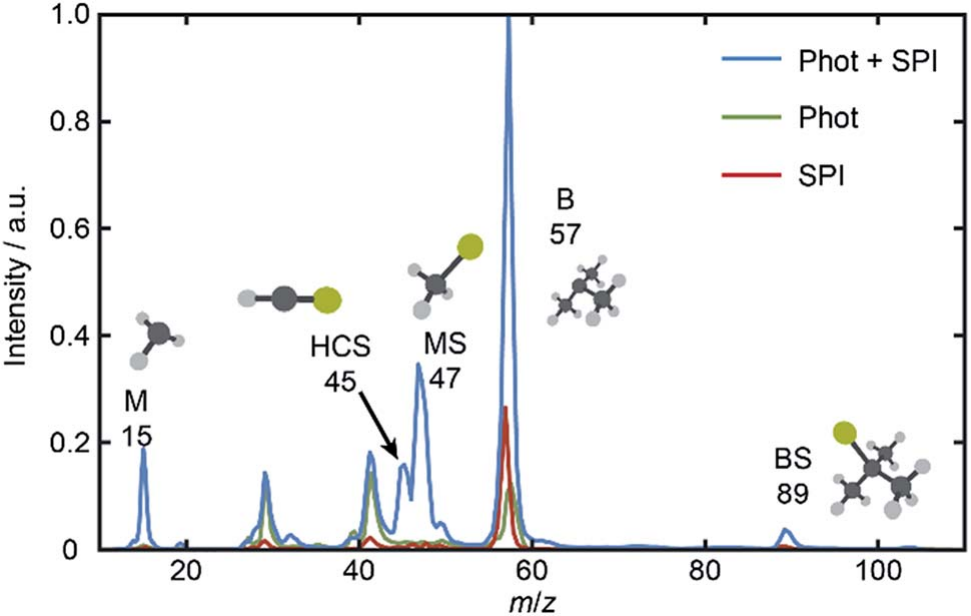
Mass spectra derived from TOF spectra measured following excitation of jet-cooled BSM with just the photolysis (*λ* = 225.0 nm) or just the SPI (*λ* = 118.2 nm) or with both laser pulses present. The parent ion (*m*/*z* 104) yield is large under all conditions, and the detector gain was therefore time-gated so as to afford maximum sensitivity only over the limited time range corresponding to *m*/*z* 10–60.

The UV absorption spectrum of BSM vapor (room temperature sample, shown in Fig. S1 of the ESI†) is less structured than that of MSM but similarly concentrated at *λ* ≤ 240 nm and attributable to the analogous 1^1^A′′ ← X[combining tilde] and 2^1^A′′ ← X[combining tilde] excitations. Fig. 1 shows a TOF mass spectrum of the fragment ions resulting from *λ* = 225.0 nm photolysis of BSM and subsequent SPI using *λ* = 118.2 nm photons, along with ‘one-color’ spectra obtained with just the photolysis or SPI laser pulse. The parent ion (*m*/*z* 104) peak is intense under all conditions and, as in previous work,^16^ the voltage applied to the MCPs was time-gated so as to provide maximum sensitivity only over limited time windows – corresponding to *m*/*z* 10–60.

**Fig. 2:**
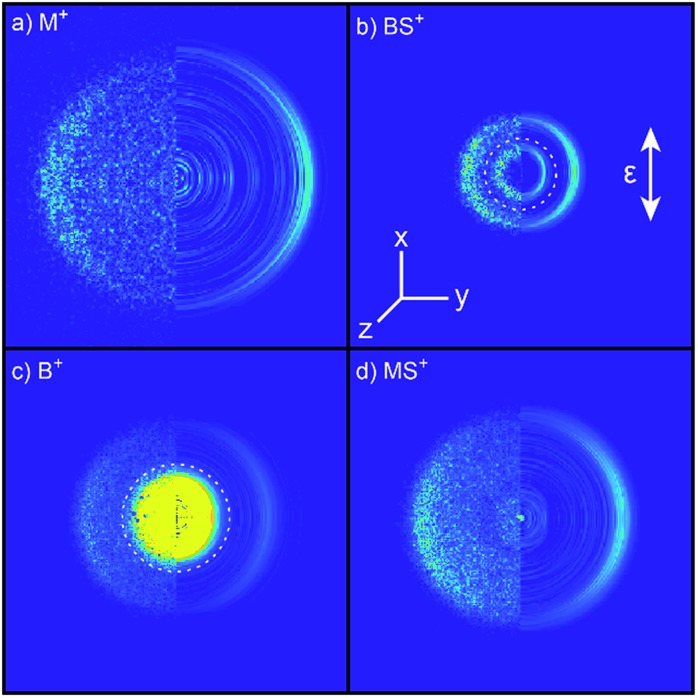
Left halves of crushed images of the *m*/*z* (a) 15 (M^+^), (b) 89 (BS^+^), (c) 57 (B^+^) and (d) 47 (MS^+^) ion signals following *λ* = 225.0 nm photolysis of BSM and subsequent SPI at *λ* = 118.2 nm, with the **ε** vector of both lasers aligned vertically in the plane of the images. The half images to the right show central slices through the corresponding symmetrized reconstructed 3-D velocity distributions. Signal inside the dashed ring superposed on panels (b) and (c) was omitted from traces demonstrating momentum matching (Fig. S3†) and the *E*_T_ traces in Fig. 3.

Inspection of such spectra reveals the parent ion peak (heavily attenuated in Fig. 1) and ‘two color’ peaks for M^+^ (*m*/*z* 15), MS^+^ (*m*/*z* 47), B^+^ (*m*/*z* 57) and BS^+^ (*m*/*z* 89) ions formed by SPI of fragments formed *via* the rival C–S bond fissions (1a) and (1b). Crushed images, obtained by summing the *x*–*y* distributions over all time bins associated with each *m*/*z* peak, are shown (left half only) in Fig. 2. The right half of each panel shows the corresponding central slice through the (symmetrized) reconstructed 3-D velocity distribution. As in the UV photodissociation of MSM,^17^–^19^ the M^+^ image shows one anisotropic ring with large radius. Similar (albeit smaller) anisotropic rings are also evident in the MS^+^, B^+^ and BS^+^ images.

The B^+^ image also contains a very intense central feature. Appearance potential data are available for various alkyl thioethers^42^ though not to the best of our knowledge for BSM. Given the substantial parent ion yield, however, it is reasonable to attribute the central feature in the B^+^ image to dissociative ionization of the BSM parent which, from Table 1, has a thermochemical threshold of ∼9.965 eV and is thus energetically feasible following absorption of either two pump photons or one probe photon. As Fig. 1 shows, there are also ‘pump’ only contributions at *m*/*z* 57, 41 and 29 which, on energetic grounds and from the form of the associated images (Table S1 and Fig. S2 in the ESI†) are most plausibly attributed to UV photodissociation of BSM^+^ parent ions – a process that could also contribute to the central feature in the pump-only B^+^ image. The inner ‘ring’ in the BS^+^ image is an artefact; the BS^+^ image also has a strong central feature (from dissociative ionization of BMS) but, under the conditions required to measure the anisotropic component clearly, appears ‘depleted’ as the centre of the detector is still recovering from being saturated by the intense B^+^ signal that shortly precedes it in the TOF-MS. We note the minimal probe-only contribution to the *m*/*z* 47 peak (Fig. 1 and 2(d)) which, again, accords with prior observations that the main fragment ion peaks in the electron impact induced mass spectrum of BSM appear at *m*/*z* 89, 57, 41 and 29.^42^Fig. 1 contains one other ‘two-color’ peak of note, with *m*/*z* 45. As in the case of MSM photolysis, this is attributable to HCS^+^ ions from dissociative ionization of primary MS products.^16^

**Fig. 3:**
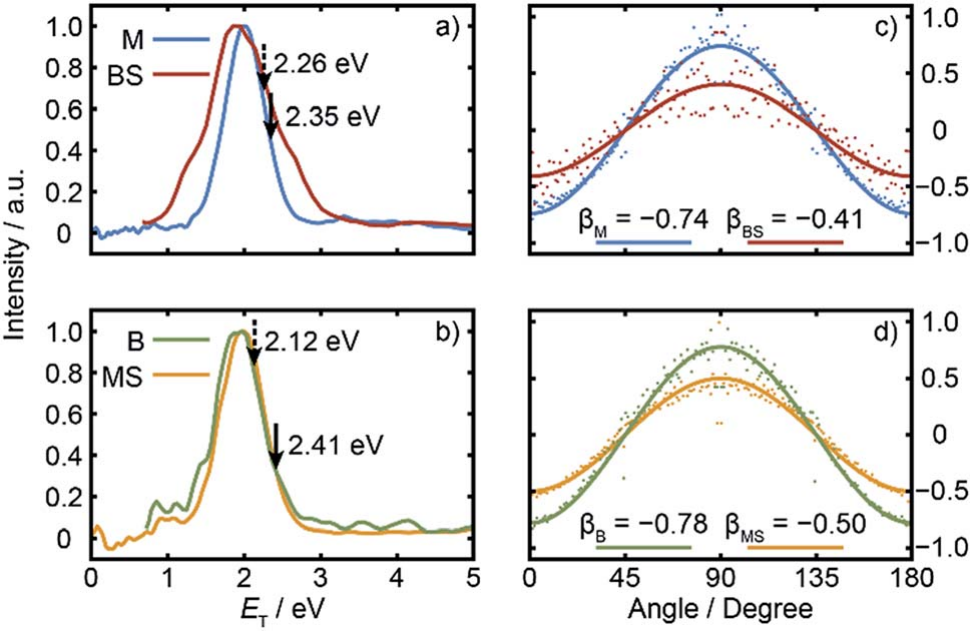
Normalised *E*_T_ distributions of the (a) M and BS and (b) B and MS fragments derived from the respective images recorded following *λ* = 225.0 nm photodissociation of BSM and subsequent SPI at *λ* = 118.2 nm. The solid and dashed arrows indicate the *E*_T(max)_ values derived using, respectively, the *ab initio* bond strengths derived in this work and the prior thermochemical data listed in Table 1. Intensity *vs. θ* plots for the (c) M and BS and (d) B and MS products plotted on common vertical scales that span the full range required for a distribution characterised by an anisotropy parameter *β* = –1, with the best-fit *β* values indicated.

Radial integration over the outer parts of the reconstructed images (*i.e.* the regions outside the dashed semi-circles in the right halves of Fig. 2(b) and (c)) yields the respective fragment velocity distributions. As Fig. S3† shows, the peaks of the momentum distributions of the partner products (*i.e.* M with BS and B with MS) match well, validating the assumption that the SPI efficiencies for each species are relatively insensitive to the particular quantum states in which they are formed. The corresponding total kinetic energy, *E*_T_, distributions derived from the M and MS data, assuming momentum matching with the respective (*i.e.* BS and B) partner fragments, are similar, as shown in Fig. 3(a) and (b). Both *P*(*E*_T_) distributions peak close to the maximum *E*_T_ values allowed by energy conservation, given a photolysis photon energy *E*_phot_ corresponding to *λ* = 225.0 nm and the thermochemical^43^–^53^ and computational data listed in Table 1, implying that most of the available energy *E*_avl_ is partitioned into product translation. We consider the differences between the respective C–S bond strengths returned by the present electronic structure calculations and those derived using available thermochemical data in Section 3.3, but this overall conclusion is not affected by these differences. As Fig. 3(a) shows, the *E*_T_ distribution derived from the image of the heaviest fragment (BS) is noticeably broader than that from the light co-fragment (M). This we attribute to space charge broadening of the heavier (and thus slower) fragment ions by the large (and relatively more proximal) yield of parent ions – a trend that was also observed – albeit less dramatically, for the heavier (*i.e.* MS) fragments formed in the UV photolysis of MSM.^16^

Fig. 3(c) and (d) show the angular distributions of the fast components of the respective M with BS and B with MS fragment distributions. All peak at *θ* = 90°, *i.e.* the fragments recoil preferentially along an axis perpendicular to the **ε** vector of the photolysis laser, as expected in the case of prompt C–S bond fission following photoexcitation involving a transition dipole moment lying perpendicular to the parent C–S–C plane. The depth of the angular modulation is fragment dependent, however, which we illustrate in Fig. 3(c) and (d) by plotting the data for each pair of partners on common vertical scales that span the full range that would be required for a distribution displaying limiting perpendicular anisotropy (*i.e.* a distribution described by an anisotropy parameter *β* = –1). The best-fit *β* values for each fragment are shown alongside the corresponding data. The alkyl fragments display greater recoil anisotropy (*β* ∼ –0.75 for both the M and B fragments) than their alkylthiyl MS and BS partners – for which the best-fit *β* parameters are, respectively, ∼–0.5 and ∼–0.4. Similar discrepancies in the *β* parameters of partner fragments were found in our SPI measurements of the M and MS fragments from UV photolysis of MSM and rationalized by showing that the photoionization probability of the alkylthiyl fragment is alignment dependent.^16^

One conclusion of this study is thus that the UV photofragmentation dynamics of BSM show clear similarities with that reported previously for MSM (and for CH_3_SH and even H_2_S). In all cases, photoexcitation involves a transition of A′′ symmetry, the photoexcited molecules dissociate by breaking either C–S (H–S) bond on a timescale that is short compared to the parent rotational period, and the resulting fragment pairs recoil with translational energies close to the upper limit allowed by energy conservation.^54^ This conclusion could have been reached using any of a number of more traditional photofragment translational spectroscopy or VMI methods, however. The prime novelty of the present study is the quantification of the relative probabilities of the rival bond fission processes.

### 3.2 Quantifying the rival C–S bond fission probabilities

**Fig. 4:**
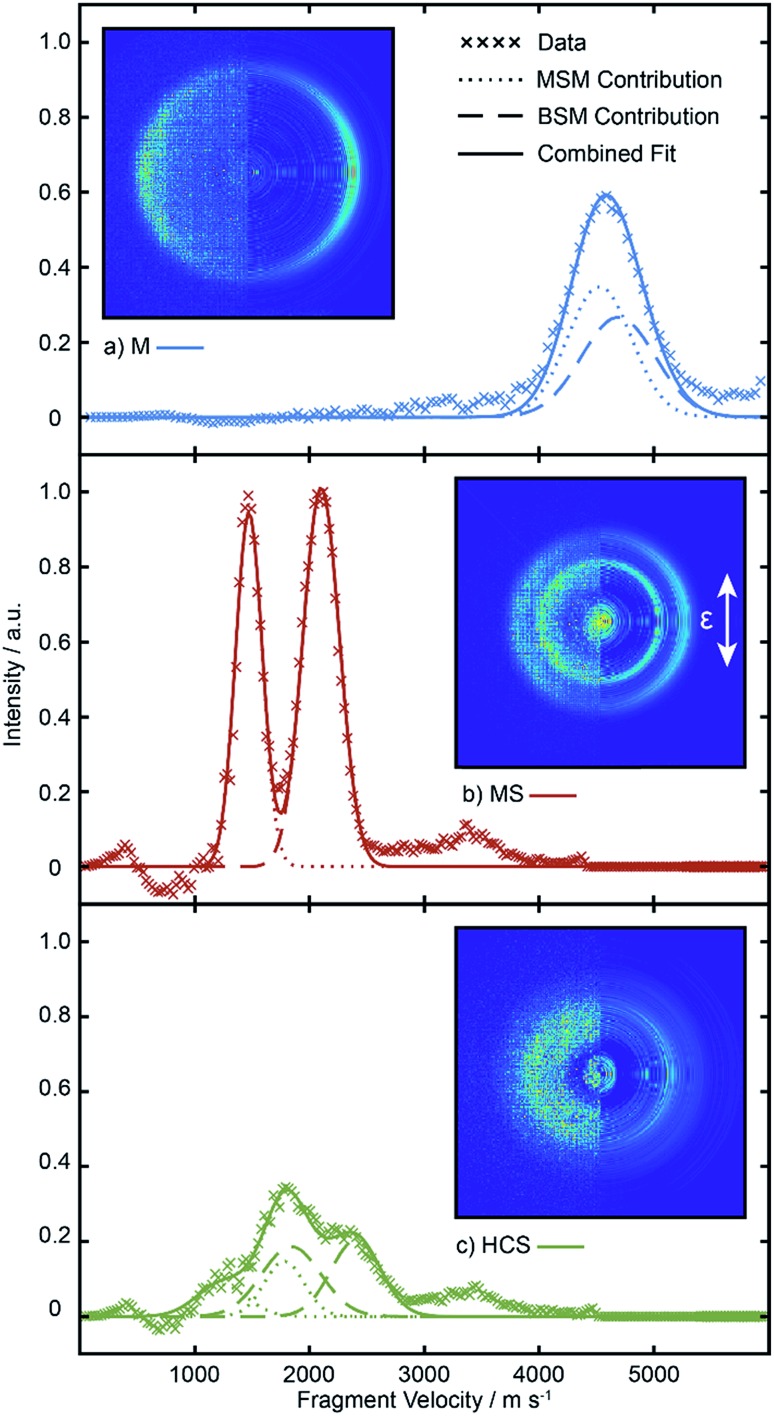
Velocity distributions derived by radial integration over the *m*/*z* (a) 15 (M^+^), (b) 47 (MS^+^) and (c) 45 (HCS^+^) ion signals following *λ* = 225.0 nm photolysis of a mixture containing equal partial pressures of BSM and MSM and subsequent SPI at *λ* = 118.2 nm, with the **ε** vector of both lasers aligned vertically in the plane of the images, along with the best-fit Gaussian functions used in determining the reported branching ratios. The left and right halves of the inset in each panel show the crushed and reconstructed central slice (after symmetrisation) images.

The left halves of the panels inset within Fig. 4 show crushed images of the (a) M^+^, (b) MS^+^ and (c) HCS^+^ fragments formed by SPI following *λ* = 225.0 nm photolysis of a jet-cooled sample containing equal partial pressures of BSM and MSM recorded under the same conditions as used for the data shown in Fig. 2. The right halves of these inset panels show the corresponding central slice through the (symmetrized) reconstructed 3-D velocity distribution. Radial integration over the reconstructed M^+^, MS^+^ (and *m*/*z* 45 HCS^+^) images yield the fragment velocity distributions shown by the points in Fig. 4(a)–(c). As in our recent MSM photolysis study,^16^ the (much smaller) *m*/*z* 45 signal is attributed to dissociative ionization of primary MS fragments and is thus treated as part of the total MS yield. The MS products from the two precursors are clearly radially separated, but the velocity distributions of the two sets of M products are too similar to be resolved.

To separate these, ion images were recorded following photolysis of pure samples of MSM and BSM. The M^+^ and SM^+^ distributions from each of these samples were reconstructed and the resulting radial distributions fitted to Gaussian functions. Note, the method is not constrained in basis set. Any appropriate fit function should work, and using the empirical distributions recorded from pure samples as basis functions works equally well in the present case (and could be preferable when attempting to account for any inhomogeneities in the detector sensitivity, or when the velocity distributions show obvious structure due to population of selected product vibronic states). The HCS^+^ distributions from the pure samples were each fitted as sums of two further Gaussians. Examples of these radial distributions (in pixel space) and the Gaussian fits for MSM, BSM and a BSM/MSM mixture following *λ* = 225.0 nm photolysis are shown in Fig. S4.† These eight Gaussian functions were used for deconvolving the contributions to the M^+^, MS^+^ and HCS^+^ radial distributions from MSM and BSM in any mixed sample. The widths and centres of all these basis functions, and the relative amplitudes of the two functions describing the HCS^+^ distribution from a given precursor at any given wavelength were all held fixed at the values listed in Tables S2 and S3 in the ESI,† and the remaining six amplitudes were allowed to float when fitting the radial distributions from photolysis of the mixtures. The best fits to each radial distribution from the mixed sample are shown by the solid lines in Fig. 4(a)–(c), with the individual contributions from the MSM and BSM basis functions shown as dotted and dashed lines, respectively.

The probabilities of B–SM and BS–M bond fission can then be derived as follows. The relative amplitudes of the contribution from each precursor in the M and MS images are given by3
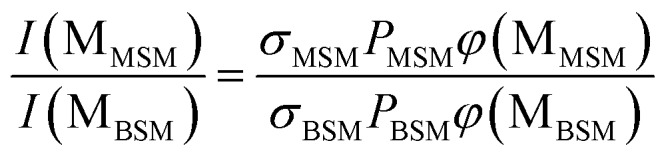
and4
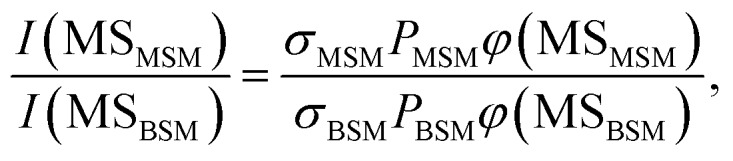
where *I*(M_MSM_) and *I*(M_BSM_), and *I*(MS_MSM_) and *I*(MS_BSM_), are the relative M^+^ and MS^+^ ion yields (with the latter including the small *m*/*z* 45 (HCS^+^) contributions), the *σ* and *P* terms are the respective parent absorption cross-sections and partial pressures, and the *φ* values are the quantum yields for forming M and MS from the respective parent molecules. The respective cross-sections for the SPI processes that convert M and MS photoproducts into the measured *m*/*z* 15 and 47 (and 45) signals are assumed to be precursor independent. Support for this assumption is provided by the quality of the inter-fragment momentum matching (see Fig. S3† and ref. 16) and the similar internal energy disposals in the given fragment from the two precursors (which, in the case of the MS fragments, is also reflected in the similar weight of the *m*/*z* 45 dissociative ionization contribution).

All these contributions are imaged simultaneously, so the ratios (3) and (4) should be independent of the photolysis and SPI laser intensities. Further, the *σ* and *P* terms are common to both ratios and all prior studies suggest that it is valid to assume *φ*(MS_MSM_) = *φ*(M_MSM_) = 1 (*i.e.* that UV excitation of MSM inevitably results in fission of one S–M bond). Combining eqn (3) and (4) then yields5
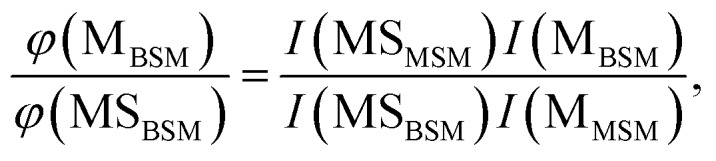
in which *all* of the quantities on the right-hand side can be determined from analysis of a *single* multi-mass imaging experiment. Further, this conclusion holds irrespective of the choice of BSM/MSM mixing ratio. The branching ratio (5) will give *absolute* branching fractions in the limit that *φ*(MS_BSM_) + *φ*(M_BSM_) = 1, which we assume in the present case given the fast dissociation timescale implied by the observed fragment recoil anisotropies. Table 2 lists the branching fractions for processes (1a) and (1b) derived from analysis of images recorded in *N* different experiments involving a range of different BSM/MSM mixing ratios, at three (closely spaced) UV wavelengths: the uncertainties on the branching ratios preclude any definitive comment about its wavelength dependence, but the data show that B–SM bond cleavage is about twice as probable as BS–M bond fission.

**Table 2:** Quantum yields for B–SM and BS–M bond fission following UV excitation of BMS. The quoted uncertainties are 2*σ* values from fitting all *N* data sets recorded (for a range of different BSM/MSM mixing ratios) at the given wavelength

	*λ*/nm		
	227.5 nm	225.0 nm	222.5 nm
*N*	21	22	21
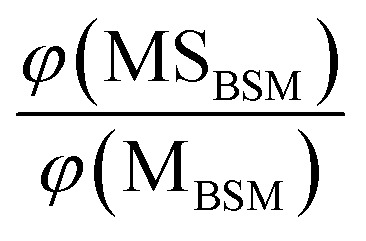	1.80 ± 0.33	2.14 ± 0.61	2.7 ± 1.3
*φ*(MS_BSM_)/%	64+4–5	68+5–8	73+7–14
*φ*(M_BSM_)/%	36+5–4	32+8–5	27+14–7

### 3.3 Rationalising the relative bond fission probabilities

**Fig. 5:**
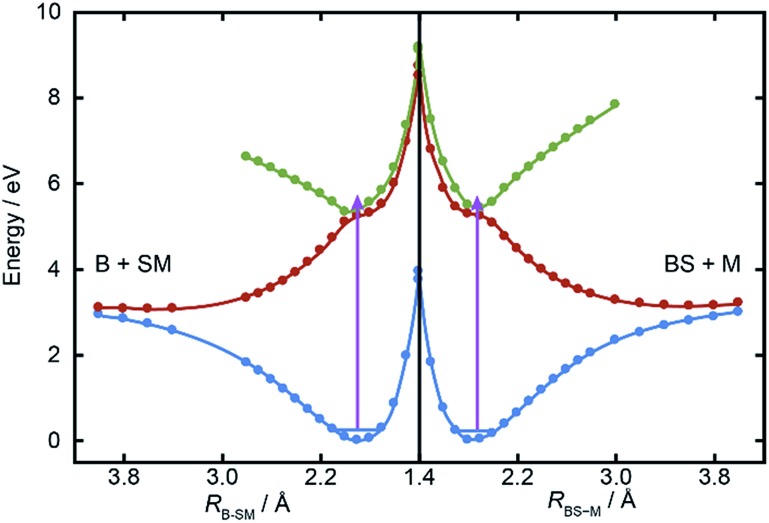
Relaxed PECs for the ground (^1^A′, in blue) and first two excited (^1^A′′, in red and green respectively) states of BSM along the *R*_B–SM_ and *R*_BS–M_ bond extension coordinates (left and right halves, respectively). The mauve arrows show the excitation energy reached by *λ* = 225.0 nm photoexcitation from the parent zero-point level.

Fig. 5 shows cuts through the adiabatic PESs for the ground and first two singlet excited states of BSM returned by the CASPT2 calculations along the B–SM and BS–M bond extension coordinates, *R*_B–SM_ and *R*_BS–M_. These were obtained by progressively extending the coordinate of interest and, at each *R* value, allowing the rest of the nuclear framework to relax to its ground state minimum energy geometry. Spin–orbit coupling, which introduces a small (tens of cm^–1^) energy splitting in the ground state MS radical,^55^ has not been included in the present calculations. As in the cases of H_2_S^27^,^28^ and MSM,^19^,^29^ the present calculations find two close-lying and interacting excited states of ^1^A′′ symmetry, the lower energy of which correlates to the B–SM and BS–M dissociation limits of current interest. As Table 1 showed, the calculated C–S bond strengths are very similar. These values are also very similar to that calculated for the C–S bond in MS–M (3.142 eV), which is in excellent accord with the experimental value (3.13 ± 0.04 eV (ref. 56)). Both calculated C–S bond strengths in BSM are slightly lower than those derived using the enthalpy of formation data collected in Table 1, and their energetic ordering is reversed. These are minor details in the context of the current work, but the *ab initio* bond strengths appear to align more sensibly with the leading edge of the *P*(*E*_T_) distributions shown in Fig. 3, suggesting that one or more of the Δ_f_*H* (0 K) values listed in Table 1 merits further attention – most likely the enthalpy of formation of the BS radical^44^,^57^ which carries a relatively large uncertainty.

The *ab initio* calculations (Fig. 5) return a ground state equilibrium B–SM bond length that is slightly longer than that for the BS–M bond: *R*_B–SM(e)_ = 1.868 Å, *cf. R*_BS–M(e)_ = 1.822 Å. The calculations also suggest that the B–SM bond is marginally the weaker bond, but it seems unlikely that either (or both) of these effects could account for the observed strong preference for B–SM bond fission. The UV absorption spectra of the lighter thioethers show diffuse structure^19^,^25^,^26^ attributable to short lived resonances supported by the higher of the ^1^A′′ PESs and any full explanation for the preferred B–SM bond fission is likely to require a much better understanding of the geometry dependences of the transition dipole moment that carries the initial photoexcitation, and of the non-adiabatic couplings that enable the subsequent bond extension.

### 3.4 Extensions and generality of the method

This study demonstrates a means of determining the quantum yields of rival bond fissions in a collision-free photolysis experiment, but also serves to illustrate several limitations:

(i) The target (T) and calibrant (C) molecules must both give measurable yields of products that report on the competing fragmentation pathways (*i.e.* M and SM in the present case). This requires that T and C must differ by just a single moiety and, in practice, that these moieties are sufficiently different (in mass) that the velocities of the reporter products from T and C can be resolved in an imaging experiment (as for the SM products in the present study). In similar vein, the current method is unlikely to be applicable when one of the rival bond fission channels yields a light (*e.g.* H) atom, since the heavy co-fragment is likely to be dwarfed by the (generally much larger) parent ion yield.

(ii) The method does not obviate the need for careful photolysis studies of both T and C in isolation, with a proper characterisation of the energy disposal within the different fragmentation channels in each case, nor for thorough checks to establish whether any alignment effects influence the detection of the respective products.

(iii) There is no *a priori* reason that all quantum states of a given product will have equal photoionization efficiencies (or equal dissociative ionization probabilities). Thus the reporter products from photolysis of T and C should ideally be formed in a similar spread of quantum states (as in the present case, and as will often be the case when comparing the photochemistries of two members of a class of homologous compounds^54^).

(iv) The analysis would also become more complicated (and less complete) if T and/or C can decay by more than just the two rival fragmentation pathways. Three-body fragmentation must become a possible decay channel upon tuning to sufficiently short photolysis wavelengths. Reference to the enthalpy of formation data listed in Table 1 suggests that three-body fragmentation of BSM is energetically possible at wavelengths below ∼200 nm. The resulting S atoms would be amenable to SPI at *λ* = 118.2 nm; their non-observation in the present study (Fig. 1) offers further validation of the assumption that BSM can only fragment by channels (1a) or (1b) at the wavelengths studied here. However, the method could still be used to provide new information (the relative yields of the rival channels giving rise to the reporter products) in cases where T and/or C can decay by more than two rival fragmentation pathways, but would not yield absolute branching fractions.

Notwithstanding these various caveats, given access to suitable SPI energies, the method should be applicable to several classes of homologous molecules that are known (or can be expected) to undergo (wavelength dependent) competitive bond fissions following UV photoexcitation. Likely examples include ethers and thioethers (such as the example demonstrated here), anisoles and thioanisoles, and ketones and thioketones.

## 4 Conclusions

This study demonstrates how, by combining ‘universal’ single photon ionization methods with recent multi-mass imaging developments (which allow simultaneous determination of the speed and angular distributions of all ionized fragments) and an internal calibrant, it is possible to quantify the relative probabilities of competing bond fission processes in a photoexcited molecule. The example chosen to demonstrate the method is the UV photolysis of an asymmetric thioether, *t*-butylmethylsulfide (BSM), at wavelengths between 227.5 nm ≥ *λ* ≥ 222.5 nm, using dimethylsulfide (MSM) as the internal calibrant. BSM dissociation yields both B + SM and BS + M products, in a ∼2 : 1 ratio. The experimental data are complemented by high level electronic structure calculations. The B–SM bond in the ground state of BSM is shown to be both slightly longer and slightly weaker than the BS–M bond, but neither difference is considered sufficient to explain the magnitude of the observed bias in favour of B–SM bond fission. We hope and expect that the present data for BMS will serve as a benchmark for testing theoretical models of excited state nuclear dynamics beyond the Born–Oppenheimer approximation and, more generally, that the study will stimulate other approaches to quantifying the absolute yields of rival bond fissions in photoexcited molecules.

## Conflicts of interest

There are no conflicts to declare.

## Supplementary Material

Supplementary information
