# Supplementary material for: Quantifying rival bond fission probabilities following photoexcitation: C–S bond fission in t-butylmethylsulfide
Source: Chem Sci. 2019 Apr 23;10(20):5290–8. doi: 10.1039/c9sc00738e (PMC6540878; doi:10.1039/c9sc00738e)
Supplement: Supplementary file 1 [file SC-010-C9SC00738E-s001.pdf]

Electronic Supplementary Material (ESI) for Chemical Science.
This journal is © The Royal Society of Chemistry 2019

Supplementary Information for

Quantifying rival bond fission probabilities following photoexcitation: C–S bond fission in *t*-butylmethylsulfide:

Matthew Bain,[1] Christopher S. Hansen,[2] Tolga N.V. Karsili [3] and
Michael N.R. Ashfold [1]

[1] School of Chemistry, University of Bristol, Bristol, U.K., BS8 1TS

[2] School of Chemistry, University of New South Wales, Sydney, NSW 2052, Australia

[3] Department of Chemistry, University of Louisiana at Lafayette, Louisiana, LA 70504, U.S.A.

Corresponding authors:

mike.ashfold@bristol.ac.uk

christopher.hansen@unsw.edu.au

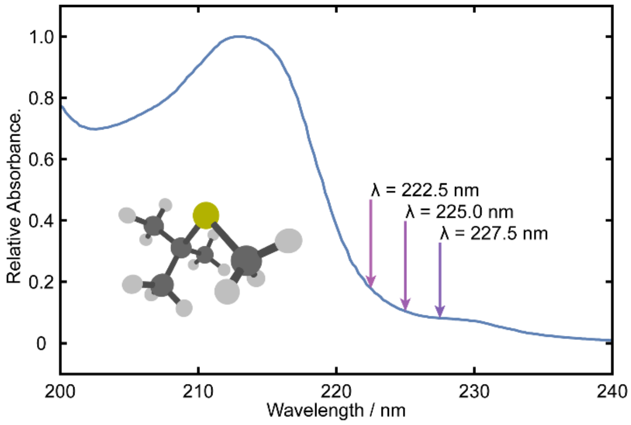

Figure S1. Absorption spectrum of the vapour above a room temperature dilute solution of BSM in *n*-hexane recorded against a background of pure *n*-hexane vapour, with the three photolysis wavelengths investigated in this study indicated by coloured arrows.

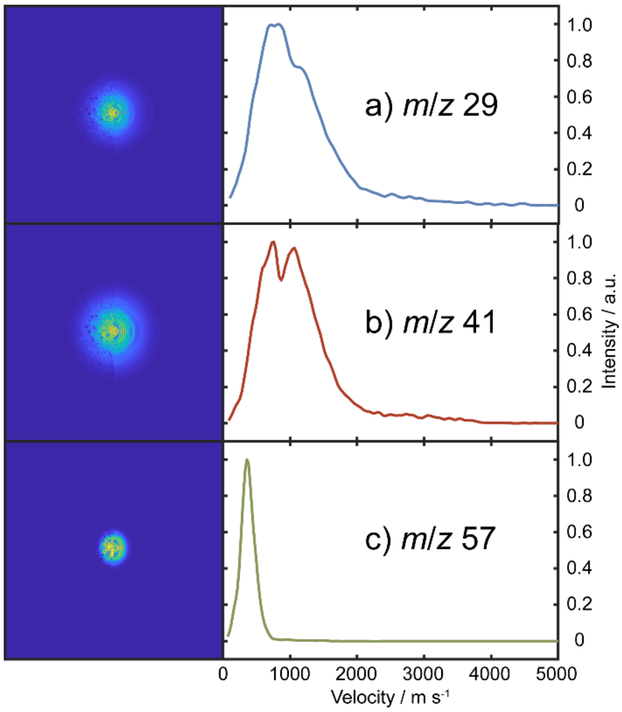

Figure S2. Images and associated velocity distributions of the *m*/*z* 29, 41 and 57 fragments formed by pump only excitation at λ = 225.0 nm.

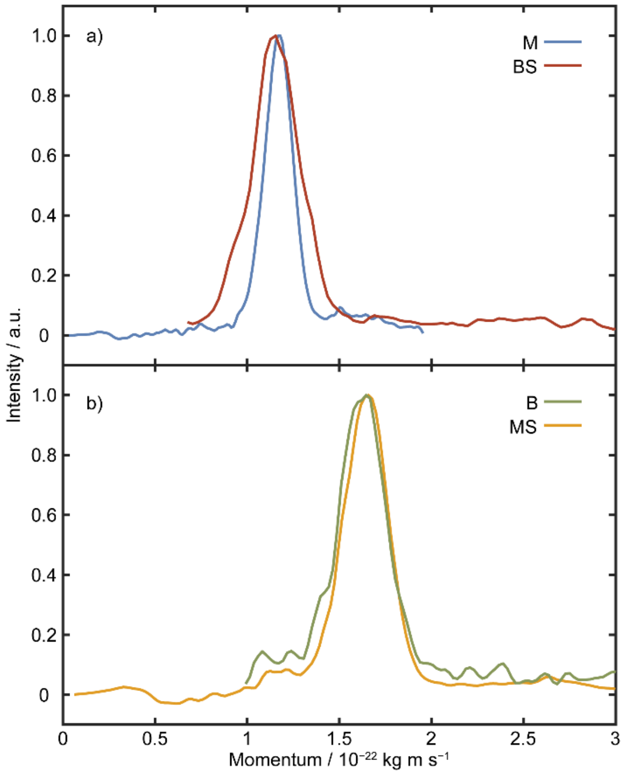

Figure S3. Normalized momentum distributions of the (a) M and BS and (b) B and MS fragments derived from images recorded following λ = 225.0 nm photodissociation of BSM and subsequent SPI at λ = 118.2 nm.

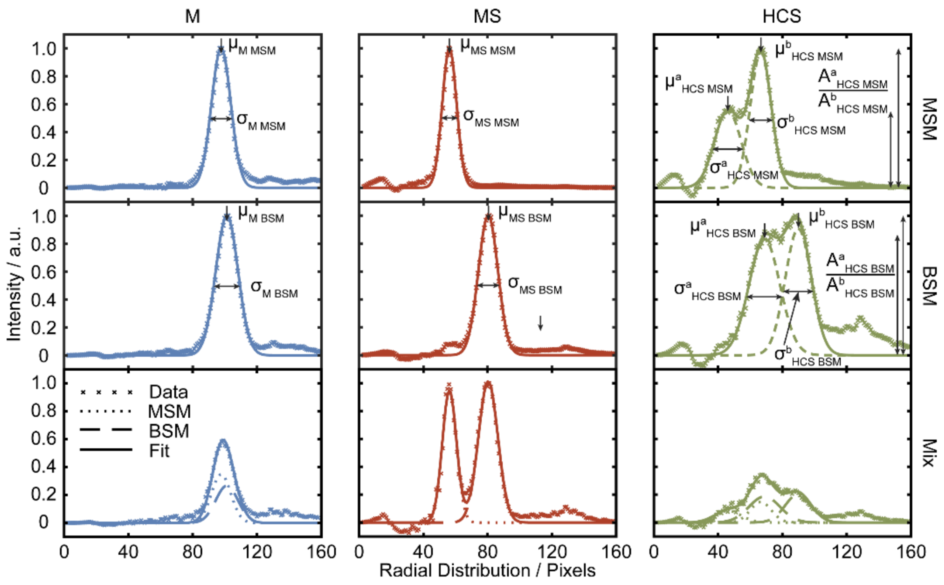

Figure S4. Radial distributions derived by integrating over the *m*/*z* 15 (M^+, left hand column), 47 (MS^+, middle column) and 45 (HCS^+, right hand column) ion signals following λ = 225.0 nm photolysis of MSM (top row), and BSM (middle row) and subsequent SPI at λ = 118.2 nm, along with the best-fit Gaussians that are used as the basis functions when fitting the corresponding distributions from BSM/MSM mixtures. The bottom row shows the fits to these distributions for the BSM/MSM mixture featured in Figure 4 of the main paper.

Species	$\Delta_f H$ (0 K)/ eV	Source
BSM	–1.257 ± 0.008	Table 1
BS	0.44 ± 0.08	Table 1
B	0.782 ± 0.007	Table 1
BSM^+	~7.15	From Table 1
B^+	7.36 ± 0.02	From Table 1
MS	1.346 ± 0.018	Table 1
$C_3H_7^+$	8.528 ± 0.003	ATcT, ref. 46 of paper
$C_3H_5^+$	9.992 ± 0.003	ATcT, ref. 46 of paper
$C_2H_5^+$	9.484 ± 0.006	ATcT, ref. 46 of paper
C_2H_5S (ES)	1.09 ± 0.08	Griller et al., ref. 44 of paper
C_3H_7S (PS)	0.78 ± 0.08	Griller et al., ref. 44 of paper
C_2H_4	0.632 ± 0.001	ATcT, ref. 46 of paper
CH_4	–0.690 ± 0.001	ATcT, ref. 46 of paper

Threshold energies (0 K)	/ eV
BSM → MS + $\mathbf{B^+}$	9.965
→ ES + $C_3H_7^+$	10.88
→ PS + $\mathbf{C_2H_5^+}$	11.52
→ MS + $\mathbf{C_3H_5^+}$ + CH_4	11.905
→ ES + $\mathbf{C_3H_5^+}$ + H_2	12.33
→ MS + $\mathbf{C_2H_5^+}$ + C_2H_4	12.72
BSM^+ → MS + $\mathbf{B^+}$	~1.55
→ ES + $C_3H_7^+$	~2.47
→ MS + $\mathbf{C_3H_5^+}$ + CH_4	~3.50
→ ES + $\mathbf{C_3H_5^+}$ + H_2	~3.95
→ MS + $\mathbf{C_2H_5^+}$ + C_2H_4	~4.25
→ PS + $\mathbf{C_2H_5^+}$	~4.40

Table S1. Enthalpies of formation of selected species (from Table 1 of main paper or the source indicated) used in estimating thermochemical threshold energies for dissociative ionization of BSM and for the photodissociation of BSM^+ cations. Fragment species detected

in the photolysis-laser-only ion TOF-MS are highlighted in bold. For reference, the energies of one and two λ = 225 nm photolysis photons are, respectively, 5.51 and 11.02 eV.

		MSM					
	λ / nm	227.5		225.0		222.5	
M^+	μ / pixels	97.3		97.7		97.9	
	σ / pixels	5.3		6.0		5.9	
MS^+	μ / pixels	55.9		56.2		56.2	
	σ / pixels	3.6		4.5		4.2	
HCS^+	μ^a, μ^b / pixels	45.2	65.6	46.0	66.5	47.0	67.4
	σ^a, σ^b / pixels	7.7	6.5	8.3	6.5	10.2	6.5
	A^a, A^b	0.56	1	0.57	1	0.72	1

Table S2. Best-fit parameters for the Gaussian functions used to describe the M^+, MS^+ and HCS^+ velocity distributions following photolysis of MSM at λ = 227.5, 225.0 and 222.5 nm with, in each case, subsequent SPI at λ = 118.2 nm.

		BSM					
	λ / nm	227.5		225.0		222.5	
M^+	μ / pixels	101.2		101.3		101.3	
	σ / pixels	6.4		6.9		8.1	
MS^+	μ / pixels	80.9		80.3		79.3	
	σ / pixels	6.0		6.1		6.4	
HCS^+	μ^a, μ^b / pixels	68.7	90.2	68.8	89.9	67.8	89.3
	σ^a, σ^b / pixels	10.6	8.5	9.7	8.1	9.8	8.3
	A^a, A^b	0.95	1	0.92	1	0.94	1

Table S3. Best-fit parameters for the Gaussian functions used to describe the M^+, MS^+ and HCS^+ velocity distributions following photolysis of BSM at λ = 227.5, 225.0 and 222.5 nm with, in each case, subsequent SPI at λ = 118.2 nm.

Species	Vibrational wavenumbers / cm^{-1}	zpe / eV
M	3282, 3282, 3103, 1403, 1403, 505	0.80
MS	3106, 3082, 3011, 1472, 1370, 1338, 866, 703, 589	0.95
B	3074, 3074, 3069, 3030, 3027, 3027, 2923, 2914, 2914, 1493, 1492, 1489, 1471, 1471, 1469, 1423, 1396, 1396, 1292, 1292, 1093, 1006, 1006, 970, 936, 936, 756, 380, 379, 259, 133, 132, 132	3.12
BS	3110, 3108, 3104, 3087, 3083, 3079, 3022, 3016, 3014, 1515, 1498, 1489, 1487, 1483, 1472, 1428, 1399, 1394, 1213, 1207, 1189, 1019, 992, 970, 934, 921, 792, 576, 385, 370, 363, 289, 274, 259, 234, 222	3.25
BSM	3122, 3117, 3106, 3104, 3099, 3088, 3081, 3077, 3038, 3026, 3019, 3015, 1518, 1504, 1503, 1491, 1489, 1483, 1479, 1467, 1430, 1401, 1401, 1355, 1240, 1236, 1193, 1049, 1040, 972, 971, 967, 938, 931, 806, 707, 570, 418, 401, 361, 315, 300, 271, 266, 225, 214, 175, 62	4.30

Table S4. Harmonic wavenumbers for the fundamental vibrational modes of the ground states of M, MS, B, BS and BSM used in establishing the zero-point corrections to the absolute and relative dissociation energies reported in Table 1 and the zero-point energies associated with the disappearing modes in the bond fissions shown in Figure 5.
